# Unveiling professional and personal preferences of early career dentists during first year of employment at the Thai dental public sector: a one-year cross-sectional study

**DOI:** 10.1186/s12903-023-03659-8

**Published:** 2023-12-09

**Authors:** Tanit Arunratanothai, Ravisorn Booncharoen, Sirapop Suwankomolkul, Nareudee Limpuangthip

**Affiliations:** 1 Faculty of Dentistry, Chulalongkorn University, 34 Henri-Dunant Road, Pathumwan, Bangkok, 10330 Thailand; 2 Department of Prosthodontics, Faculty of Dentistry, Chulalongkorn University, 34 Henri-Dunant Road, Pathumwan, Bangkok, 10330 Thailand

**Keywords:** Compulsory service, Dentist allocation, Health policy, Health system, Health workforce, Policy implementation

## Abstract

**Background:**

Despite the implementation of various government policies to retain Thai dentists in public sector, a high turnover rate among early career dentists has persisted for decades. This study aims to explore factors relating to early career dentists’ choice of the public sector as their preferred workplace and decisions relating to staying, resigning, or relocating from the workplace after one-year employment.

**Methods:**

A one-year cross-sectional survey was conducted among Thai early career dentists who began working in 2020 using two sets of online questionnaires. The first survey assessed factors influencing dentists’ decision to choose the public sector as their preferred workplace at the beginning of the year. The second survey investigated factors influencing dentists’ decision to stay, resign, or relocate from the same workplace at one-year after employment. Descriptive statistics and multivariable binary logistic regression were used for data analysis.

**Results:**

A total of 198 early career dentists completed the online survey questionnaire at the starting point (December 2020–January 2021), and 186 dentists completed the one-year employment questionnaire. The living environment and provided amenities and facilities were the most influential factors in their decision to choose and remain in the public sector. Conversely, their attitude toward unrelated job descriptions and an increased opportunity to pursue postgraduate studies were the most relevant factors when deciding to relocate to a new workplace. Factors such as delayed authority in bureaucracy, hometown location, and being in relationship status were the most significant contributors to resignation from the public sector.

**Conclusions:**

The major factors influencing dentists’ choice and retention in the public sector include the living environment, supportive supervisors and colleagues, and the availability of opportunities for further postgraduate education. Meanwhile, factors impacting retention after one year of work are related to hometown location and the bureaucracy system. Collaborations among ministries, tailored to each local community’s specific requirements, may enhance dentists’ retention in public sectors.

**Supplementary Information:**

The online version contains supplementary material available at 10.1186/s12903-023-03659-8.

## Background

Health workforce is one of the six components within the building blocks of a health care system [1]. A society with skilled individuals in the healthcare workforce will possess valuable resources that will promote a broader healthcare coverage and drive socioeconomic prosperity. Therefore, the management of human resources is essential in all clinical disciplines including dentistry. Since the last century, the Thai government has implemented various policies to manage the dental workforce across the country. Early policies by the Ministry of Public Health (MoPH) were focused on speeding up the graduation of new dentists to develop the first dental public services and to scale up the hiring of dentists in rural clinical settings [2–4].

Dental education in Thailand commenced with the establishment of the Faculty of Dentistry at Chulalongkorn University in 1940 [5]. Thailand currently has 18 dental faculties, both public and private, producing approximately 600–900 graduate dentists annually for the last decade [6]. The undergraduate dental program lasts for 6 years receiving Doctor of Dental Surgery (DDS) degree. Postgraduate dental education in Thailand includes 5 distinct certificate programs, enrolling approximately 100–200 individuals each year. These programs encompass Doctor of Philosophy, Master of Science, Residency Training, Higher Graduate Diploma in Clinical Sciences, and Graduate Diploma in Clinical Sciences programs, offering a range of specialized training and research opportunities in the field of dentistry [7]. Tuition fees for undergraduate students in Thailand significantly differ between public and private dental faculties. For instance, at Chulalongkorn University, a public dental school, the estimated tuition cost throughout the undergraduate program is approximately 1 million Thai Baht (THB) (≈ 27,070 USD as per October 2023) [8]. In contrast, at a private dental school, such as Rangsit University, the total expenses, including tuition and equipment fees, amount to around 6 million THB [9]. The students at public dental school receive subsidies for a portion of their tuition and equipment fees, provided by either the government or the university itself.

Since 1982 until the present, the MoPH also launched a policy to prevent the brain drain of Thai dentists both from public to private clinical settings and from Thailand to foreign countries. Under this recent policy, all undergraduate students from public dental schools receiving government support for tuition must sign an employment contract. According to this contract, Thai dentists are required to undergo a three-year compulsory service (CS) in a designated province within Thailand after graduating from dental schools [10]. Currently, there are two main routes of CS in public sector, comprising working as a civil servant under the MoPH and being employed by another public sector as a state enterprise employee. If there is a contract breach, recent graduates must pay a fine up to 400,000 Thai baht (≈ 10,830 USD) [11].

To access the hospitals under the MoPH, the dentists must undergo an allocation system run by drawing and casting lots to distribute recent dental graduates to each province. Meanwhile, to access the other entities of public sector, the dentists will utilize a direct application to compete the selection by the organization [12]. Most civil servant dentists work in primary- to secondary-level hospitals, primarily providing dental services within their designated areas, and handling additional tasks as assigned. Some civil servant dentists in Provincial Public Health Offices focus on administrative work alongside dental services. Meanwhile, the roles of dentists as state enterprise employees can vary depending on the organization [13]. For instance, those in dental schools often engage in further studies to enhance their academic qualifications and capabilities, while those in other organizations primarily provide dental services to personnel, even if it is not directly affiliated with the Ministry of Public Health. Both civil servant and state enterprise employees work in public hospitals under different institutions, leading to varying benefit packages, although there is income similarity. Dentists working in public sector, including those fulfilling CS, are not restricted to offering dental services solely in public hospitals but are also allowed to work in private dental clinics or hospitals. However, this work typically occurs outside regular government working hours [14].

During the CS period, the early career dentists receive a monthly salary of approximately 18,000 THB (≈ 490 USD), totaling around 40,000–50,000 THB per month (≈ 1,080–1,350 USD). In contrast, dentists in the private sector earn their income based on the number of patients they treat, typically a minimum of around 3,000 THB (≈ 80 USD) per day. It is noted that the standard average monthly salary for bachelor degree in public institutions in Thailand is started at 15,000 THB (≈ 400 USD) per month [15]. To retain the dentists in the public sector, the government launched and continuously implemented a policy to improve the financial welfare of dentists in rural areas since 1987 until the present date [16–21].

Many cross-sectional surveys have assessed the satisfaction level from the allocated dental graduate [22–26]. Residing near to their hometown, gaining knowledge and experience, and having opportunities to pursue studies in their favorite dental specialty were the main positive factors for choosing employment at the public sector. However, there is a mismatch between their expected monthly income and the reality of total incomes from the public sector. Despite all efforts from the government to promote Thai dentist retention in public hospitals across Thailand, early career dentists still resign from the healthcare system at a rate of more than 50% during the completion of the three-year CS [14, 27]. The objective of this study is to explore factors relating to early career dentists’ choice of the public sector as their preferred workplace and decisions related to stay, resign, or relocate from the workplace after one-year employment.

.

## Methods

### Study design and ethical approval

The present study had a cross-sectional design and included early career Thai dentists at the start of their first employment in the public sector. The data was collected at two time points, upon undergraduate graduation (December 2020 to January 2021) and one-year after employment (December 2021 to January 2022). The eligible participants were newly graduated Thai dentists who started their career in public sector in 2020. Dentists who did not completely fill the questionnaires and those who pay the CS program–related fine were excluded from the study. The early career dentists who paid the CS program-related fine were excluded because our study aimed to track resignations after one year, and this group would not be eligible for this one-year follow-up. The study had been performed in accordance with the Declaration of Helsinki, and was approved by the Human Research Ethics Committee of the Faculty of Dentistry, Chulalongkorn University (code no. HREC-DCU 2020−110, approved on December 4th, 2020).

### Sample size calculation

The sample size determination followed specific criteria when the real population falls under 1,000 subjects, which defines the estimated sample size to be at a minimum of 15% of this population [28]. There was a target population of 719 graduated dentists from public dental schools and registered by the Dental Council of Thailand in 2020, and this was defined as the entire population under study. The sample size was at 108 (15% of the 719 registered dentists).

### Online questionnaires

Two questionnaires were designed to survey the dentists at the beginning of employment and one-year after employment. Content validity of the questionnaire was evaluated by 5 senior Thai dentists who had been working in academic institutions and public hospitals for more than 5 years. Face validity was conducted via a pilot study on 10 dentists who started their work in 2019. After receiving feedback from the pilot study, a consensus was reached between the investigators and experts. The two final versions of the online questionnaire were developed via an online Google Form application (Google LLC, California, Delaware US). Test-retest reliability was assessed for the first questionnaire with a sample of 10 dentists who began working in 2019 and for the second questionnaire with a sample of 10 dentists who started in 2018. The questionnaires were distributed through online group which aggregated all enrolled graduated dentists who started their employment in 2020 and via a representative from the 10 public dental schools in Thailand.

For the first data collection at the beginning of employment, a questionnaire was employed, containing 5 parts as follows (Supplementary Fig. 1):

- Part I: Consent and acceptance form for participating in the research survey.
- Part II: Personal demographic data, comprising of age, sex, marital status, admission track, graduated dental school, planning for postgraduate education, type of CS in public sector.
- Part III to V: List of reasons relating to dentists’ decision on choosing the workplace. Each part targeted early career dentists working in the public sector, selected through 3 different methods: drawing lots to become civil servants in public hospitals under the MoPH, direct application for state enterprise employee positions under other entities of public sector, and appointment as civil servants in predetermined areas through a quota system. The participants used a four-point ordinal scale to express their perception of the level of impact. Additionally, the study surveyed the dentists’ determination regarding whether to continue working in the public sector.

At the second data collection time point (one-year after employment in 2021), a questionnaire contained 5 parts as described below (Supplementary Fig. 2):

- Part I: Consent and acceptance form for participating in the research survey.
- Part II: Personal demographic data, comprising of age, sex, marital status, admission track, graduated dental school, planning for postgraduate education, type of CS and current status of their CS in public sector.
- Part III to V: List of reasons relating to dentists’ decision after one-year working. Each part focused on the dentists undergoing 3 different scenarios: those who remained at the same workplace since 2020, those who relocated to other hospitals, and those who resigned from CS. They were further categorized as resignation and stay/relocate. The participants used a four-point ordinal scale to express their perception of the level of impact.

### Statistical analysis

Data were analyzed using the SPSS program, version 28.0 (IBM, Chicago, Illinois US). Descriptive statistics were conducted to determine the percentage distribution (%) of categorical variables and to calculate the mean and standard deviation (SD) for continuous variables. Percentage distributions of the factors relating to dentists’ decision in choosing and retaining in the workplace at the beginning and one-year after employment were calculated. Two multivariable binary logistic regression models were conducted to investigate the individual dentist-related factors associated with initial application for state enterprise employment, and resignation after one-year employment.

## Results

**Fig. 1:**
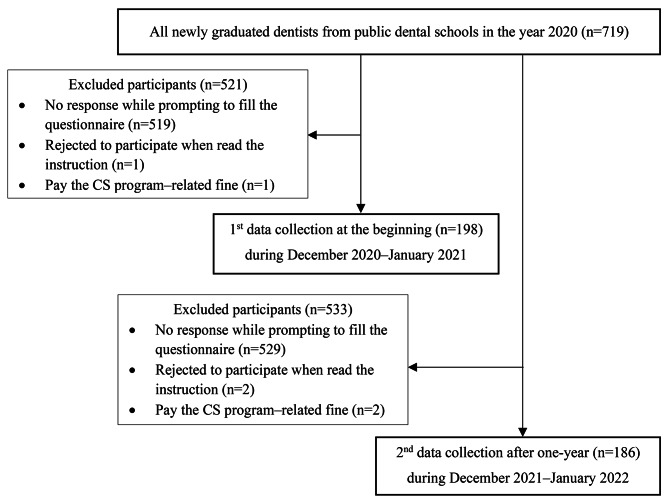
Flowchart with the study design and sample size of participants

Figure 1 demonstrates flow diagram of the study design and sample size of participants. A total of 198 and 186 dentists completed the survey at the beginning and after one-year employment, calculated as 27.5% and 25.9% of the population, respectively. The participants were averagely 25.2 ± 1.2 years in both years (Table 1). Following graduation, nearly all participants chose to work in hospitals under the MoPH, which was assigned through drawing lot as the allocation system (83.0–84.9%). A smaller percentage worked in other entities of public sector through direct application (12.1–14.4%). Only a minority worked in predetermined workplaces through a quota admission track.

**Table 1:** Characteristics of early career dentists at the beginning and one-year after employment

Variables	At the beginning (year 2020, n = 198)		After one-year employment (year 2021, n = 186)	
	n	percentage (%)	n	percentage (%)
Sex:				
Male	57	28.8	51	27.4
Female	141	71.2	135	72.6
Marital status:				
Single	137	69.2	123	66.2
In relationship	60	30.3	62	33.3
Married	1	0.5	1	0.5
Region of dental school:				
Central	128	64.7	98	52.7
Northern	39	19.7	62	33.3
North-eastern	23	11.6	7	3.8
Southern	8	4.0	19	10.2
Admission track:				
The Consortium of Thai Medical Schools (COTMES)	81	40.9	75	40.4
Central Admission	51	25.8	49	26.3
Increased Rural Dentist Production Project	58	29.3	57	30.6
Chula Rural	6	3.0	2	1.1
Project under Ministry of Interior	2	1.0	3	1.6
Having plan to higher level of graduation:				
Yes	100	50.5	139	74.7
No	98	49.5	47	25.3
Distribution of graduated dentist to workplace:				
Institutions under the MoPH applied through drawing lot	169	85.4	156	83.9
Other public sectors through direct application	24	12.1	27	14.5
Predetermined workplace from quota project	5	2.5	3	1.6
Decision after one-year employment:				
Staying at the same workplace			163	87.6
Moving to other workplaces	N/A		9	4.8
Resigning from compulsory service			14	7.6

MoPH, Ministry of Public Health; N/A, not applicable

**Fig. 2:**
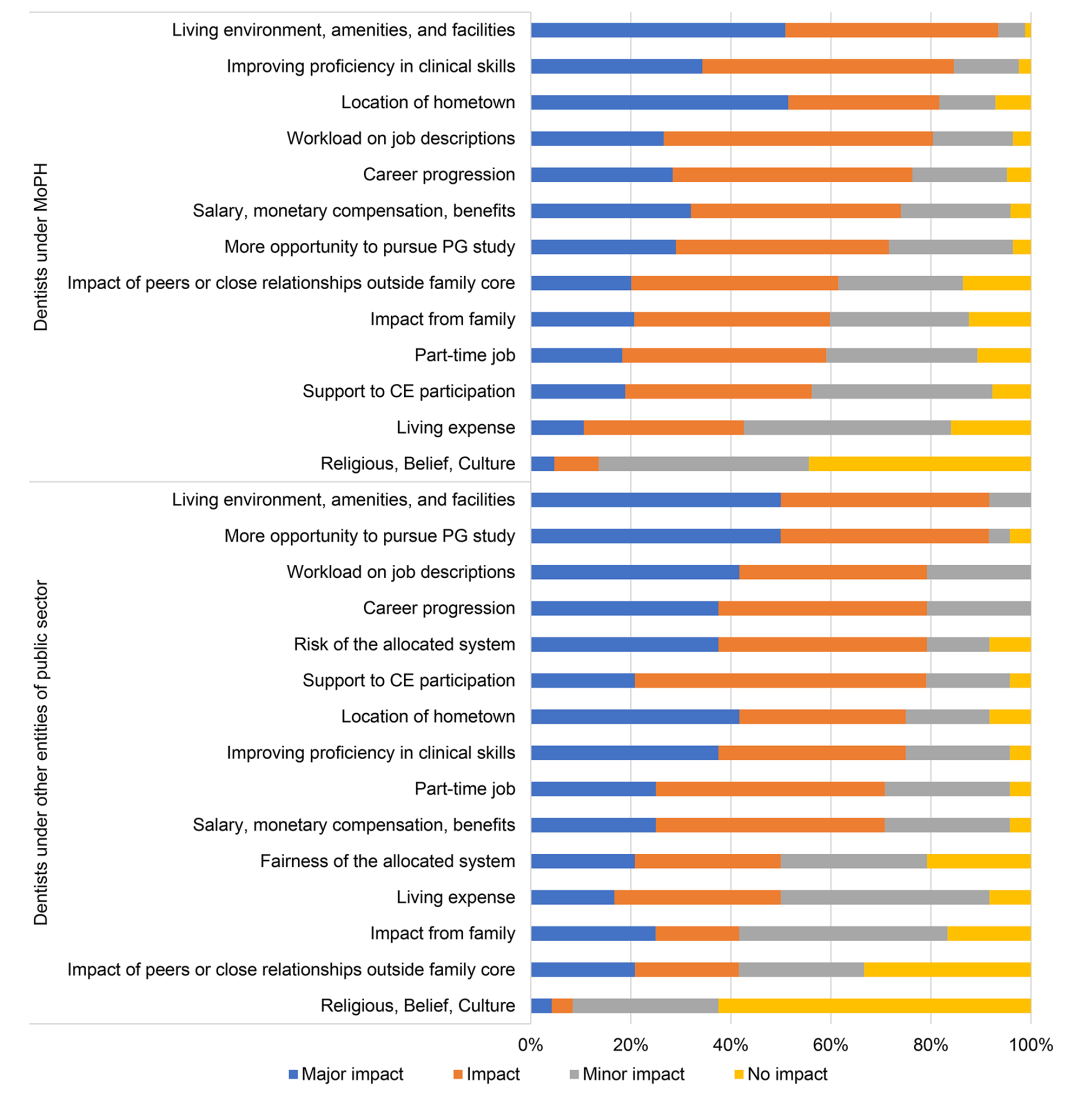
Factors related with the early career dentists’ decision to undergo CS under the MoPH and other entities of public sector at starting the employment (n = 198)

Figure 2 illustrates the reasons relating to individuals’ decision when choosing their workplace, specifically between hospitals under the MoPH and other entities of public sector. The results show that the most common reasons for both groups were living environment, amenities, and facilities (93.5% and 91.7%, respectively), closely followed by a clear job description (80.4% and 79.2%, respectively). Among those working under the MoPH, other significant factors relating to their decision included the opportunity to enhance proficiency in clinical skills (84.6%) and hometown location (81.7%). On the other hand, for individuals working in other entities of public sector, the opportunity to pursue postgraduate education was a major reason, with a percentage of 91.7%. Religious, beliefs, and culture had the least impact on the decision-making to choose and stay in their workplace for both groups. Around 30–40% of the participants chose not to continue working in public hospitals permanently, while approximately 10–20% expressed a desire to continue their employment in public hospitals (Table 2).

**Table 2:** Dentists’ determination whether to continue working in public sector according to type of compulsory service in public sector at the beginning of employment (n = 198)

Dentists’ determination	Total	Hospitals under the MoPH (n = 169)	Other entities of public sector with direct applicable (n = 24)	Predetermine workplace with quota admission track (n = 5)
Forever	35	32 (18.9)	2 (8.3)	1 (20.0)
Not forever	58	47 (27.8)	9 (37.5)	2 (40.0)
Have not decided	105	90 (53.3)	13 (54.2)	2 (40.0)

**Fig. 3:**
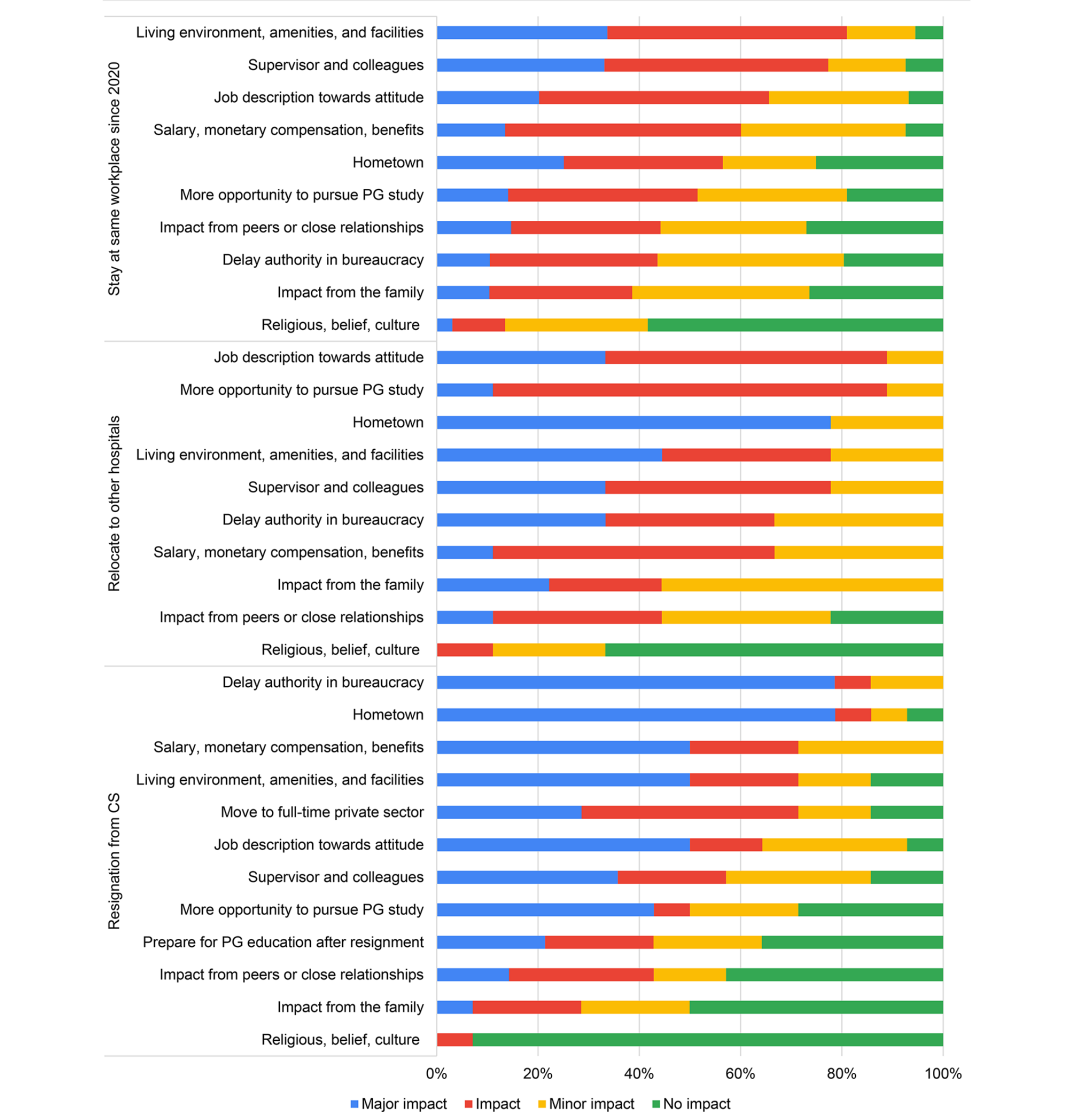
Factors related with the early career dentists’ decision who stayed at the same workplace, relocated to other hospitals, or resignation from CS (n = 186)

After one-year employment, most participants (86.7%) continued working at the same workplace under the MoPH. A small percentage relocated to other public workplaces (4.8%) or resigned from the public sector (7.4%). The most common reasons for their decision to continue working at the same workplace were living environment, amenities and facilities, as illustrated in Fig. 3. In contrast, for the dentists who relocated to other public workplaces, the most common reasons impacting their decision were the mismatch between their job description and desired requirements, as well as the desire for further postgraduate education (88.9%). For those who resigned from public sector, the primary reasons mentioned were the desire to return their hometown location and issues related to the delay authority in bureaucratic system (85.7%).

Taking individual factors into account, early career dentists in relationships were more prone to undergo direct admission at the beginning of their employment year and to resign early from the CS system after one-year employment (Table 3). Furthermore, individuals with plans for postgraduate education showed higher odds of selecting direct application for state enterprise employment compared with the drawing-lots selection for civil servant positions.

**Table 3:** Multivariable binary logistic regressions of the associations between individual dentist-related factors and initial direct application for state enterprise employment (n = 198), as well as resignation after one-year employment (n = 186)

Dentist-related factors	At the beginning: Direct admission	One-year after employment: Resignation
	(ref = drawing lots)	(ref = stay and relocate)
	OR (95% CI)	OR (95% CI)
Age (years)	0.92 (0.49, 1.73)	0.77 (0.41, 1.47)
Sex:		
Male	1 (ref)	1 (ref)
Female	0.98 (0.32, 3.02)	0.74 (0.17, 3.20)
University:		
Bangkok	1 (ref)	1 (ref)
Central	2.51 (0.24, 20.1)	0.08 (0.01, 1.83)
North	5.59 (0.31, 10.9)	0.29 (0.02, 5.56)
Northeast	1.65 (0.17, 16.0)	0.37 (0.05, 2.74)
South	0.70 (0.11, 4.54)	0.04 (0.01, 2.71)
Status:		
Single	1 (ref)	1 (ref)
In relationship/Married	3.45 (1.30, 9.44)*	9.54 (1.27, 10.2)*
Admission track:		
COTMES	1 (ref)	1 (ref)
Central admission	0.71 (0.21, 2.32)	2.44 (0.09, 34.1)
Chula rural and others	N/A	3.22 (0.08, 20.9)
Planning for postgraduate education:		
No	1 (ref)	1 (ref)
Yes	8.02 (2.25, 28.6)*	1.48 (0.39, 5.67)

*Significant association at *p* < 0.05. N/A, not applicable because early career dentists from the rural admission track had to work in the predetermined area

## Discussion

To enhance dentist retention in the public healthcare system, the government should implement policies that cater to the needs of early career dentists. The living environment, amenities, and facilities are major reasons that influence early career dentists to choose and retain in the public sector. Additionally, the location of their hometown and the presence of all delay processes of authority in bureaucracy affect the dentist retention under the MoPH. Moreover, the availability of opportunities for further postgraduate education and the presence of partnerships also impacts dentists’ decisions to choose their working on other entities of public sector.

Our findings indicated that the primary consideration for early career dentists in choosing their workplace is the living environment, amenities, and facilities, both at the beginning of their career and after one-year employment. When comparing between hospitals under the MoPH and other public sector healthcare, MoPH hospitals are primarily located in rural areas with limited urbanization. In contrast, institutions like universities offer superior facilities and resources. To retain dentists in the public sector, particularly under the MoPH, the government should prioritize key aspects such as infrastructure development across all regions, providing clean water and electricity in workplace areas, offering standardized and safe housing accommodations, and facilitating convenient transportation with an efficient mass transit system connecting rural to major urban centers.

The location of dentists’ hometown significantly influences their decisions to choose or resign under the MoPH. Interestingly, dentists who initially chose their workplaces through direct application did not prioritize their hometown location. However, after one year of employment, our study revealed that 85.7% of dentists cited hometown location as the main reason for resignation. This decision to return to their hometown may be driven by a desire to live in a familiar environment from childhood to adolescence. Additionally, we observed that the delay authority in bureaucracy was another major reason for resignation, with a similar percentage to the hometown’s location. This suggests that the hometown’s location may overshadow the true underlying reasons behind dentists’ resignations, such as misalignment with attitudes towards local community healthcare and the impact of globalization in the digital era.

In Thailand, the hometown location plays a pivotal role in influencing dentists’ resignations. To address this, a quota admission track is employed to recruit students from rural areas into dental schools, ensuring that they return to their hometowns as dentists upon graduation [29]. Dentists graduating from the quota admission track are individuals from specified rural areas who are granted the privilege to study in dental schools without having to compete with students nationwide for admission. However, after completing their education, only 40% of them intended to continue working in their hometown community. This percentage is consistent with regular dental graduates who did not receive special admission privileges to dental schools. Thus, a thorough evaluation of the quota’s effectiveness is essential to assess its outcomes and appropriateness. Alternatively, implementing more restrictive contracts with these students in special projects could increase retention within this group. Moreover, the government faces challenges in providing positions for all dentists who signed contracts in the quota program, which mandates their return to serve in their hometown locations to fulfill their obligations.

Our study highlights that postgraduate education opportunities have major influence on dentists’ decisions to apply for work as a state enterprise employee in other public sectors. The chance for specialized training in further postgraduate education is also an important factor influencing dentists’ decisions to relocate or resign. In Thailand, public hospitals under the MoPH offer limited seats for dentists to undergo residency training programs as an encouragement to retain them in the public healthcare system. These opportunities are also restricted to specialized branches facing shortages within the hospitals under the MoPH. Meanwhile, the specialized training seats are more often reserved for dentists affiliated with the public sector [30]. As a result, some early career dentists choose to apply directly to other public sector entities, such as standard- to advanced-level hospitals, which often require dental specialists compared to hospitals under MoPH, encompassing primary- to secondary-level hospitals. Dentists aspiring for guaranteed study in their desired specialized field relocate to other hospitals to secure a quota or even resign from their current positions to pursue their studies. It is essential to consider whether the exclusive allocation of specialized training quotas to certain hospitals positively contributes to retaining dentists within the public healthcare system. However, continuing education (CE) or short courses training does not influence dentists’ decisions regarding their workplace or resignation.

The living environment, amenities, facilities, opportunities for postgraduate studies, hometown locations, and delays in bureaucracy influence dentists’ decisions. As a result, some dentists may choose to apply directly to work in the public sector, where they can consider familiar factors and avoid the uncertainties associated with the allocation system, in which the MoPH assigns dentists to workplaces through drawing lots.

While the salary or monetary compensation may not meet the dentists’ expectations, relying on increased salary or monetary compensation to prevent resignations may not be feasible long-term strategy, as the government has continuously announced this approach since 1987 until now [16, 18–20, 17]. Salary and monetary compensation increases had been implemented to match the expected minimum level of monthly income [23]. Dentists do not prioritize salary and compensation among their top three reasons for resigning, indicating that these factors have less influence on their decision. Additionally, living expenses do not significantly impact dentists’ choices of their workplace in the public sector, affecting less than 50% of the dentists. This may be attributed to the relatively low cost of living in Thailand, exemplified by Bangkok, a representative city, when compared to other countries reported by the World Bank [31]. Therefore, implementing financial policy could lead to an inefficient allocation of the government’s annual budget.

During the early stages of their careers, dentists’ decisions to resign are minimally influenced by factors such as family and close relationships outside the family core [32]. This may be because, during the initial stages of their careers, dentists focus on gaining their work experience to pave the way for their career progression in the near future. However, after three-year of CS, family and close relationships outside the family core may begin to exert influence on their work decisions, aligning with the stages of Erikson’s psychosocial development [33]. Although it is possible that a child taking over a private dental clinic from their parents might impact dentists’ decisions to resign, there is currently insufficient empirical evidence to confirm this hypothesis. From our findings, all the resigned dentists who were in relationships reported hometown as the most influential reason for their decision-making. This suggests that they may have plans to return to their hometowns to settle down and start families.

Dentists around the age of 25 to 26 may experience a quarter-life crisis phenomenon [34]. Therefore, the dentists who continue working in their original locations require supporting factors to sustain in their practice. Supportive supervisors and colleagues are crucial in providing emotional and technical assistance to early career dentists, serving as professional role models and alleviating stress and anxiety in the dental workplace. This support is vital for increasing job satisfaction among the Y, Z, and Millennial generations of dentists [35, 36].

Based on our findings, we highlight that factors influencing decisions regarding CS and resignation are integral to a fulfilling life, aligning with Maslow’s hierarchy of needs [37]. Addressing the manpower issue in the healthcare workforce requires a comprehensive approach that extends beyond healthcare measures alone, necessitating collaboration across various ministries. The CS implementation for early career dentists, with a three-year contract and penalties for contract breaches, aims to foster commitment and co-responsibility in serving the public sector. However, it may not effectively establish a strong connection between dentists and the local community because dissatisfaction can be resolved by paying the fine to free oneself from government regulations. Policymakers must conduct a thorough evaluation of the long-term benefits and drawbacks of the three-year CS policy to make informed decisions.

Some limitations in this study includes a short-term follow-up and focusing only on dentists within the public sector. Additionally, the low response rate could introduce non-response bias. It is possible that dentists with a negative view of the CS system, such as those who did not receive what they had expected or were dissatisfied with the application process, may have been less willing to respond to the survey. Further research is required to undertake in-depth investigations into the reasons why some early career dentists decide to pay the CS program-related fine rather than undergoing the CS could provide valuable insights for addressing individual limitations and concerns. We recommend policymakers to conduct long-term follow-up with dentists from various health system sectors and generations to develop comprehensive strategies that can effectively motivate dentists to work in dental public practices. This approach aims to enhance dentist retention in rural public health care facilities over the long term, benefiting both dentists’ personal and professional levels. Identifying hospitals with successful dentist retention can provide valuable insights for adapting this model to diverse hospital contexts, respecting, and valuing the unique needs of each local community.

## Conclusion

Major factors relating to dentists choosing and retaining in the public sectors included the living environment, supportive supervisors and colleagues, as well as the availability of opportunities for further postgraduate education. Meanwhile, retention of dentist after one-year employment were related with hometown location and the bureaucracy system. Collaborations among ministries, tailored to each local community’s requirements, may enhance the dentists’ retention in public sectors.

### Electronic supplementary material

Below is the link to the electronic supplementary material.

Supplementary Material 1

## Data Availability

Dataset generated during the current study is available upon request to the corresponding authors.
