# Supplementary material for: Unveiling professional and personal preferences of early career dentists during first year of employment at the Thai dental public sector: a one-year cross-sectional study
Source: BMC Oral Health. 2023 Dec 9;23:989. doi: 10.1186/s12903-023-03659-8 (PMC10710705; doi:10.1186/s12903-023-03659-8)
Supplement: Supplementary file 1 — Supplementary Material 1 [file 12903_2023_3659_MOESM1_ESM.docx]

**Supplementary Figures**

**Supplementary figure 1: Questionnaire structure to survey at starting of employment (2020)**

The instruction and accepted or rejected the study

Part 1

Part 2

Personal Information

- Age
- Sex
- Graduated dental school (DDS)
- Marriage Status
- Admission track (DDS)
- Plan to postgraduate study
- Type of CS in public sector
- Contact (E-mail)

Part 3

Part 4

Part 5

For dentists who attended CS as quota in admission track with predetermined workplace

For dentists who attended CS as direct the state enterprise employee

For dentists who attended CS as civil servant under MPH

**Supplementary figure 2: Questionnaire structure to survey at one-year after employment**

The instruction and accepted or rejected the study

Part 1

Personal Information

- Age
- Sex
- Graduated dental school (DDS)
- Marriage Status
- Current status in CS (2021)
- Admission track (DDS)
- Plan to postgraduate study
- Type of CS in public sector
- Contact (E-mail)

Part 2

Part 3

Part 4

For dentists who resigned from CS

For dentists who stayed at the same workplace in CS since 2020

Part 5

For dentists who relocated to other hospitals in CS
